# Developmental plasticity of mitochondrial function in American alligators, *Alligator mississippiensis*

**DOI:** 10.1152/ajpregu.00107.2016

**Published:** 2016-10-05

**Authors:** Gina L. J. Galli, Janna Crossley, Ruth M. Elsey, Edward M. Dzialowski, Holly A. Shiels, Dane A. Crossley

**Affiliations:** ^1^Faculty of Medical and Human Sciences, University of Manchester, Manchester, United Kingdom;; ^2^Faculty of Life Sciences, University of Manchester, Manchester, United Kingdom;; ^3^Developmental Integrative Biology Research Group, Department of Biological Sciences, University of North Texas, Denton, Texas; and; ^4^Rockefeller Wildlife Refuge, Grand Chenier, Louisiana

**Keywords:** developmental plasticity, reptile, hypoxia, heart, mitochondria, ectotherm

## Abstract

The effect of hypoxia on cellular metabolism is well documented in adult vertebrates, but information is entirely lacking for embryonic organisms. The effect of hypoxia on embryonic physiology is particularly interesting, as metabolic responses during development may have life-long consequences, due to developmental plasticity. To this end, we investigated the effects of chronic developmental hypoxia on cardiac mitochondrial function in embryonic and juvenile American alligators (*Alligator mississippiensis*). Alligator eggs were incubated in 21% or 10% oxygen from 20 to 90% of embryonic development. Embryos were either harvested at 90% development or allowed to hatch and then reared in 21% oxygen for 3 yr. Ventricular mitochondria were isolated from embryonic/juvenile alligator hearts. Mitochondrial respiration and enzymatic activities of electron transport chain complexes were measured with a microrespirometer and spectrophotometer, respectively. Developmental hypoxia induced growth restriction and increased relative heart mass, and this phenotype persisted into juvenile life. Embryonic mitochondrial function was not affected by developmental hypoxia, but at the juvenile life stage, animals from hypoxic incubations had lower levels of Leak respiration and higher respiratory control ratios, which is indicative of enhanced mitochondrial efficiency. Our results suggest developmental hypoxia can have life-long consequences for alligator morphology and metabolic function. Further investigations are necessary to reveal the adaptive significance of the enhanced mitochondrial efficiency in the hypoxic phenotype.

the majority of ATP in oxidative tissues is derived from the process of oxidative phosphorylation (OXPHOS) in the mitochondrion (for a review see 57). The proton gradient that drives ATP synthesis is generated by the transfer of electrons along the respiratory electron transport chain (ETC) where oxygen acts as the terminal electron acceptor from cytochrome *c* oxidase (COX, Complex IV). Despite the high affinity of COX for oxygen [P50 as low as 0.01 kPa (29)], maximum respiration (and therefore ATP production) can be limited by environmental hypoxia if mitochondrial oxygen tension falls below ~1 kPa (29). In the absence of any compensatory mechanisms, ATP demand in hypoxic cells outstrips ATP supply, and essential ATP-dependent processes fail, toxic waste products accumulate, and necrotic/apoptotic processes are activated (for a review see 64).

Because of the fundamental effects of hypoxia on cellular metabolism, organisms that inhabit hypoxic environments have evolved a suite of adaptations to maintain ATP balance at low oxygen tensions (61). In the short term, animals can make physiological adjustments that improve oxygen extraction from the environment, such as increasing ventilation rate, redistributing blood flow to hypoxia-sensitive organs, and improving hemoglobin oxygen-binding characteristics (53, 62). However, extensive periods of hypoxia require a more robust response to maintain energy balance and avoid the accumulation of toxic waste products. Biochemical and molecular strategies that permit long-term hypoxic survival have been extensively studied in hypoxia-tolerant organisms (for reviews see 19, 37–40, 44, 61, 66). Several strategies are commonly utilized, including metabolic rate suppression, the activation of ATP-producing pathways that are oxygen independent, and the inhibition of pathways associated with aerobic metabolism, such as electron transport and reactive oxygen species (ROS) production (41, 53, 61). In particular, structural and functional remodeling of the mitochondria has emerged as a critical component of hypoxic survival (24, 53, 55). Mitochondrial volume density has been observed to decrease with chronic hypoxia in a range of adult vertebrates, and this is often associated with large-scale reductions in ETC Complex activities and aerobic capacity (15, 21, 24, 25, 33, 34, 42, 55, 56). Mitochondrial efficiency can be improved by CIV subunit switching which maximizes the yield of ATP per oxygen molecule consumed and minimizes the production of harmful ROS (23). Last, ROS production can be further curtailed during chronic hypoxia by downregulation of uncoupling proteins which mildly uncouple the mitochondrial membrane potential (48).

While the effects of chronic hypoxia on mitochondrial function are well documented in adult vertebrates, similar information is almost entirely lacking for earlier life stages. The effect of hypoxia on embryonic physiology is particularly interesting, as metabolic responses during development may have lifelong consequences. In contrast to reversible responses during juvenile or adult life, plastic responses during development can permanently alter an organism’s structure, function, and behavior (52). The new phenotype remains largely fixed after maturity and can even be transmitted to subsequent generations (5). This phenomenon, known as developmental plasticity, is particularly relevant to vertebrates that develop in hypoxic environments.

Many reptiles are oviparous and bury their eggs in subterranean nests. Field studies and laboratory simulations have shown initial oxygen tensions in newly laid reptilian nests are ambient (21% oxygen) and remain at this level for ~2 wk (2, 49). However, hypoxia often develops in reptilian nests as a result of the combined changes in gas conductance, rising egg mass metabolism, and metabolic activity of nest microorganisms (1, 7). The extent of hypoxia is nest dependent, but estimates of oxygen tensions range between 11 and 20% oxygen in some species (1). Given the variability in reptilian nest oxygen tensions, we hypothesized that variable oxygen levels would produce different mitochondrial phenotypes and enzyme activities that best support heart function at the oxygen concentration experienced during development. Furthermore, we expected that the different mitochondrial phenotypes would prevail into juvenile life (developmental plasticity). To this end, we investigated the effects of chronic developmental hypoxia on cardiac mitochondrial function in embryonic and juvenile American alligators (*Alligator mississippiensis*). We have chosen this species and tissue-type because American alligators naturally develop in hypoxic environments and developmental hypoxia is known to increase relative cardiac mass in embryonic alligators (12, 13, 17), pointing towards underlying metabolic adjustments. Studying the interplay between hypoxia and alligator ontogeny will improve our understanding of the role that the environment plays in shaping phenotypic variation.

## METHODS

### 

#### Animals.

American alligator eggs (*Alligator mississippiensis*; *n* = 73 embryos from 10 clutches) were collected from wild nests at the Rockefeller Wildlife Refuge in Grand Chenier, LA. To accurately establish the initial percentage of incubation, two eggs from each clutch were used for staging according to Ref. 20 (total incubation period: 72 days at 30°C). Eggs were weighed, numbered, and transported by car to the laboratory. The eggs were transported in the original nest media to protect the developing animal from vibrations (which can increase mortality) and to maintain the micro environment during transit. Embryos were incubated at 30°C in a walk-in, constant-temperature room (Percival Scientific, Perry, IA), ensuring that all embryos were developing as females. All embryos were incubated in plastic containers containing a 1:1, vermiculite:water mixture. Water content of the vermiculite, determined by mass at the beginning of incubation, was maintained by weighing the box twice weekly, with water added as needed.

Embryos were allocated into two oxygen treatment groups; 10% oxygen (*n* = 39) and 21% oxygen (*n* = 34). Ten percent O_2_ is the low value used in previous studies and slightly exceeds the ~85 Torr (11.3 kPa) measured in American crocodile nests [*Crocodylus acutus* (49)]. While we acknowledge that there is no “normal” oxygen tension for developing alligators, 10% and 21% oxygen groups will be designated “hypoxic” and “normoxic,” respectively, for the purposes of this manuscript. Oxygen incubations began at ~20% of development. Egg containers were sealed inside large Ziplock bags, and two holes in the bags allowed parallel inflow and outflow of gas. Oxygen mixtures were set using rotameters downstream of compressed N_2_ and air or air alone, with mixtures passing through a H_2_O-bubbler to ensure adequate water saturation of ≥80–95% relative humidity. Gas composition was monitored continuously with an oxygen analyzer (S-3AI, Ametek Applied Electrochemistry). Embryos were either harvested at 90% development (*n* = 56), reflected as in ovo developmental stages as “late” 27/“early” 28, based on Ferguson (20; also see 13), or transferred to 21% oxygen, allowed to hatch, and reared for up to 3 yr at 21% oxygen and 24°C (*n* = 17). All studies were approved by The University of Northern Texas (UNT) animal ethics committee IACUC 11-007.

#### Isolation of mitochondria.

Alligators were euthanized with intravenous injection of 100 mg/kg pentobarbital. Body and heart mass were measured in all animals, egg mass was measured in embryos, and snout-vent length and head size were measured in juveniles. Because of the small heart size of embryonic alligators, 4 animals were used (4 hearts pooled) to liberate enough tissue (~400 mg) for mitochondrial isolation. Mitochondria from embryonic and juvenile alligator hearts were isolated by adapting previously published protocols (26). The hearts were dissected free of connective tissue and rinsed with ice-cold homogenization buffer (250 mM sucrose, 10 mM HEPES, 1 mM EGTA, 1% fatty-acid-free BSA, pH 7.4 at 4°C) and then minced into small pieces in ice cold buffer. The tissue suspension was homogenized using three passes of a loose-fitting Teflon pestle at 100 rpm in a 12-ml glass mortar, and the homogenate was centrifuged at 600 *g* for 10 min at 4°C in polycarbonate centrifuge tubes. The supernatant was then removed, filtered through cheesecloth, and centrifuged again at 9,000 *g* for 10 min at 4°C. The supernatant was removed and discarded, and the resulting pellet was washed with fresh buffer to remove the light “fluffy” layer of the pellet (considered to be damaged mitochondria). The dark pellet was then resuspended in 12 ml of buffer and centrifuged again at 9,000 *g* for 10 min at 4°C. Finally, the pellet was resuspended in 200 µl of fresh buffer and immediately analyzed for protein content by the Bradford technique. The mitochondrial suspension was kept on ice until assayed.

#### Respiration of mitochondria.

Mitochondrial respiration was measured with an Oroboros Oxygraph 2-k high-resolution respirometry system (Oroboros Instruments, Innsbruck, Austria). Oxygen electrodes were calibrated daily with air-saturated respiration solution (in mM: 0.5 EGTA, 1.4 MgCl_2_, 20 taurine, 10 KH_2_PO_4_, 20 HEPES, 1% BSA, 60 K-MES, 110 sucrose, pH 7.1, adjusted with 5 N KOH). Zero calibrations were achieved by injecting yeast into the experimental chambers. Oxygen solubility in the assay medium was calculated as described previously (31). Two identical respiration chambers (chamber A and chamber B) held at the same temperature were run in parallel for each experimental run. Isolated mitochondria (0.45–0.60 mg protein/ml) were added to each chamber containing 2 ml of respiration medium. All measurements of respiration rates were carried out at the embryos incubation temperature (30°C).

#### Protocol for determining maximum respiration rates through individual ETC Complexes.

Three parameters are commonly used to assess mitochondrial function (for reviews, see 10, 59). First, OXPHOS (otherwise known as State III respiration) is the maximum rate of mitochondrial ADP-stimulated respiration, and is a measure of mitochondrial ATP production. Second, Leak respiration rate (otherwise known as State IV respiration) represents mitochondrial oxygen consumption that occurs in the absence of ATP generation, mainly to compensate for proton leak across the mitochondrial inner membrane. Since proton leak accounts for 20–25% of basal metabolic rate in vertebrates, a reduction in this parameter represents a significant energy-saving mechanism. Last, the respiratory control ratio (RCR, calculated here as OXPHOS/Leak state) provides a measure of the degree of coupling between oxidation and phosphorylation, or in other words, the efficiency of mitochondrial ATP production.

**Fig. 1.**
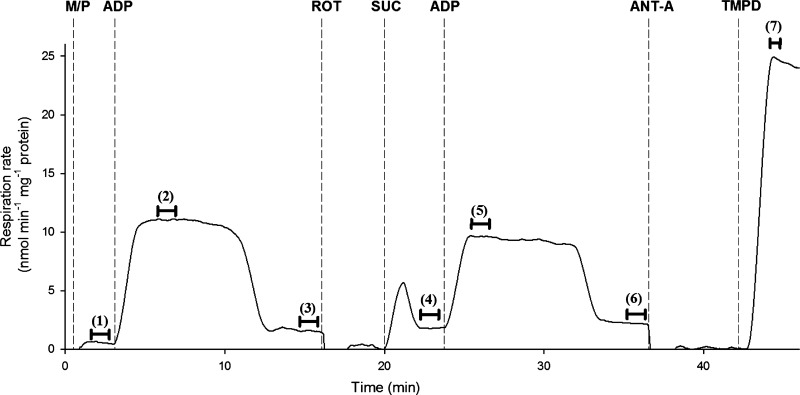
Representative trace of experimental protocol. Original trace from juvenile alligator mitochondria showing the experimental protocol used to measure mitochondrial respiratory function (see methods for full protocol details). Dotted lines signify substrates/inhibitors injected, as follows: M/P, malate and pyruvate; ADP, adenosine diphosphate; ROT, rotenone; SUC, succinate; ANT-A, Antimycin-A; TMPD, *N*,*N*,*N*′,*N*′-tetramethyl-*p*-phenylenediamine dihydrochloride. Black bars indicate time period where an averaged measurement was taken, as follows: *1*) Leak_N_ CI-CIV, *2*) OXPHOS CI-CIV, *3*) Leak_T_ CI-CIV, *4*) Leak_N_ CII-CIV, *5*) OXPHOS CII-CIV, *6*) Leak_T_ CII-CIV, and *7*) OXPHOS CIV.

OXPHOS, Leak respiration rate, and RCR were measured in the presence of Complex I substrates pyruvate and malate (electron transfer through Complexes I–IV) or the Complex II substrate succinate combined with the Complex I inhibitor rotenone (electron transfer through Complexes II–IV). Additionally, respiratory flux with electron transfer through Complex IV alone was measured via the addition of the electron donor tetramethyl-*p*-phenylene-diamine (TMPD). The protocol used to measure these parameters was adapted (from 45, 59) and is shown in Fig. 1. Briefly, pyruvate (5 mmol/l) and malate (0.25 mmol/l) were used as a carbon substrate and to spark the citric acid cycle, respectively. Under these conditions, mitochondria are in Leak respiration in the absence of adenylates (Leak_N_). OXPHOS with electron transfer through Complexes I–IV was achieved through addition of saturating levels of ADP (250 µmol/l) and a second Leak respiration state was measured following complete ADP phosphorylation (Leak_T_). Leak_N_, OXPHOS, and Leak_T_ were then measured with electron transfer through Complexes II–IV by inhibiting Complex I with rotenone (0.5 μmol/l) and adding succinate (10 mmol/l) followed by ADP. Antimycin A (5 μmol/l) was then added to block Complex III, and flux through Complex IV was assessed by adding the electron donor tetramethyl-*p*-phenylene-diamine (TMPD, 0.5 mmol/l). To avoid oxidation of TMPD, ascorbate was added before TMPD injection. In a separate set of experiments, juvenile mitochondria respiring with malate/pyruvate and saturating levels of ADP were allowed to enter Leak_T_ state. Oligomycin (1 µg/ml) was then injected to induce a third Leak respiration state, Leak_Omy_, where ATP recycling through contaminating ATPases cannot contribute and respiration reflects intrinsic uncoupling (59).

#### Enzyme assays.

The activities of citrate synthase and ETC Complexes I, II, IV, and V were measured in frozen ventricle. Briefly, ~80–100 mg of frozen tissue was weighed into a 2-ml tube and diluted 1:5 in homogenization buffer (for citrate synthase, the buffer contained 5 mM EDTA, 0.1% Triton X and 50 mM HEPES, pH 7.4; for Complex I–V: 25 mmol/l K_2_HPO_4_, 5 mmol/l MgCl_2_, pH 7.2). The tissue was homogenized with a bullet blender (Averill Park, NY) at speed 6 for 4 min. For Complexes I–V, the resulting homogenate was then centrifuged at 600 *g* for 10 min, and the supernatant was retained and centrifuged again at 600 *g* for 10 min. For citrate synthase, the resulting homogenate was centrifuged once at 10,000 *g* for 2 min. In both cases, the supernatant from each sample was immediately frozen at −80°C and all assays were performed on the first thaw. The maximal activity (*V*_max_) was determined with a spectrophotometer at 25°C with a VersaMax spectrophotometer (Molecular Devices, Sunnyvale, CA) in assay buffer. For Complexes I–V the assay buffer contained 25 mmol/l K_2_HPO_4_, 5 mmol/l MgCl_2_, 100 mmol/l KCl, and 2.5 mg/ml BSA, pH 7.2. For citrate synthase, the assay buffer contained 50 mM Tris·HCl, pH 8.0.

Rotenone-sensitive Complex I activity was monitored as a reduction of 5,5′-dithiobis(2-nitrobenzoic acid) (DCIP) at 600 nm over 5 min (assay buffer with 100 μmol/l DCIP, 0.2 mmol/l NADH, 65 μmol/l ubiquinone 2, 2 μg/ml antimycin A) in the absence or presence of 2 μg/ml rotenone. Complex II was also monitored as the reduction of DCIP over 5 min (assay buffer with 100 μmol/l DCIP, 20 mmol/l succinate, 65 μmol/l ubiquinone 2, 2 μg/ml antimycin A, and 2 μg/ml rotenone). For Complex IV activity, oxidation of reduced cytochrome *c* was monitored at 550 nm for 3 min (assay buffer with 0.6 mmol/l lauryl maltoside and 50 μmol/l reduced cytochrome *c*). Cytochrome *c* was reduced by ascorbate in 50 mmol/l Tris (pH 8.0) and the ascorbate was then removed by dialysis. Oligomycin-sensitive Complex V activity was measured as oxidation of NADH (340 nm) for 5 min (5 mmol/l ATP, 2 mmol/l PEP, 0.2 mmol/l NADH, 3 U/ml lactate dehydrogenase, and 3 U/ml pyruvate kinase) in the absence or presence of 0.5 μg/ml oligomycin. Citrate synthase activity was monitored in the presence or absence of oxaloacetate by the appearance of 5-thio-2-nitrobenzoic acid as a result of the reaction of free acetyl-CoA with 5,5′-dithiobis(2-nitrobenzoic acid) at 412 nm over a 10-min incubation period (assay buffer with 0.5 mM oxaloacetate, 0.3 mM acetyl-CoA, 0.15 mM 5,5-dithiobis-2-nitrobenzoic acid). Extinction coefficients were empirically determined to quantify *V*_max_ values for each assay. Enzyme activities were normalized to total soluble protein, which was quantified according to Bradford (7a).

#### Calculations and statistics.

All reported mitochondrial respiration rates are corrected for residual oxygen consumption (nonmitochondrial respiration measured in the presence of antimycin A) and normalized to milligram of dry protein. Respiratory control ratios (RCRs) were calculated as the ratio of saturating ADP concentrations (OXPHOS), and the respiration rate after all ADP had been phosphorylated (Leak_T_). The ratio of ADP phosphorylated to atoms of oxygen consumed (P/O ratio) was calculated by making linear extrapolations of oxygen concentration in the OXPHOS state after ADP addition, and at Leak_T_ after ADP depletion. The difference in oxygen concentrations at the intercepts is the total oxygen uptake (30). Statistical significance between oxygen exposure groups and ontological stages were assessed with a one- or two-factor repeated-measures ANOVA, as appropriate, and Student–Newman–Keuls post hoc tests. Statistical tests were performed using SigmaStat software (version 4; Systat Software, San Jose, CA). All data are reported as means ± SE.

## RESULTS

### 

#### Animal biometry and baseline mitochondrial function.

**Fig. 2.**
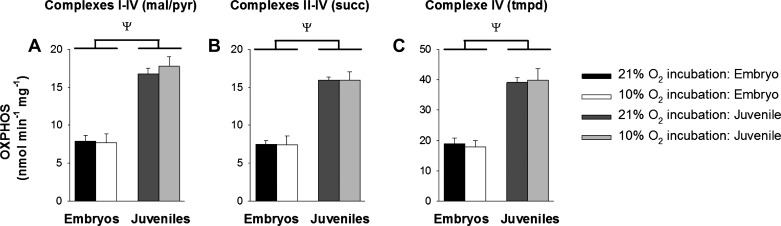
Effect of ontogeny and developmental hypoxia on OXPHOS. Mitochondria were isolated from alligator embryos exposed to 21% and 10% oxygen during development (black and white bars, respectively) and their juvenile counterparts (dark and light gray bars, respectively) subsequently raised in 21% oxygen. OXPHOS was measured with different substrates: Malate and pyruvate (electron transport through Complexes I–IV, mal/pyr, *A*), succinate (Complexes II–IV, succ, *B*), and tetramethyl-*p*-phenylenediamine (Complex IV, tmpd, *C*). Data are means ± SE. ψSignificant difference between embryos and adults (2-way ANOVA). *P* < 0.05; *n* = 8 and 6 for 21% and 10% oxygen-exposed adults, respectively, and 5 for embryos.

**Fig. 3.**
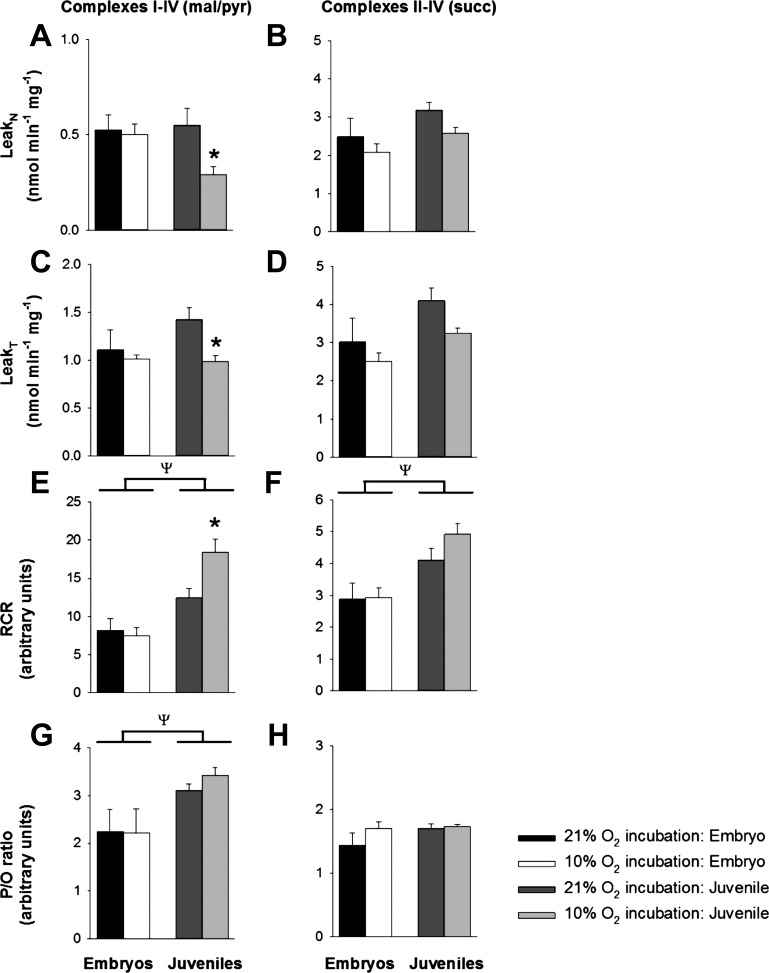
Effect of ontogeny and developmental hypoxia on Leak respiration states and mitochondrial efficiency. Mitochondria were isolated from alligator embryos exposed to 21% and 10% oxygen during development (black and white bars, respectively) and their juvenile counterparts (dark and light gray bars, respectively) subsequently raised in 21% oxygen. Mitochondria were either given malate and pyruvate as substrates (electron transport through Complexes I–IV, mal/pyr, *left panels*), or succinate in the presence of rotenone (Complexes II–IV, succ, *right panels*). Parameters: leak respiration in the absence of ADP (Leak_N_, *A* and *B*), leak respiration after phosphorylation of ADP to ATP (Leak_T_, *C* and *D*), the respiratory control ratio (RCR, *E* and *F*) and the phosphate/oxygen ratio (P/O, *G* and *H*). Data are means ± SE *Significant difference between 21% and 10% oxygen exposure groups, and ψsignificant difference between embryos and adults (2-way ANOVA). *P* < 0.05, *n* = 8 and 6 for 21% and 10% oxygen-exposed adults, respectively, and 5 for embryos.

Exposure to developmental hypoxia significantly reduced body mass and increased the heart-to-body weight ratio of embryonic alligators at 90% development and this effect persisted into juvenile life (Table 1). Embryonic and juvenile cardiac mitochondria isolated from both oxygen exposure groups were in excellent condition, as attested by high RCRs (RCR = 7–18 with malate/pyruvate and 3–5 with succinate, see Fig. 3, *E* and *F*). In all experimental groups, OXPHOS differed depending on which Complexes were involved with electron transfer, in the order of Complexes II–IV (succinate as substrate) < Complexes I–IV (pyruvate and malate as substrates) < Complex IV (TMPD as an electron donor) (Fig. 2).

**Table 1.** Effect of developmental hypoxia on embryonic and juvenile alligator morphometrics

	Egg Mass, g	Embryo Mass, g	Heart Mass, g	Heart-to-Body Mass, g/g
Embryos				
Incubated at 21% oxygen (*n* = 7)	69.3 ± 4.6	33.5 ± 2.4	0.11 ± 0.01	0.0031 ± 0.0002
Incubated at 10% oxygen (*n* = 7)	69.7 ± 4.3	23.0 ± 1.4*	0.09 ± 0.01	0.0040 ± 0.0002*

	Body Mass, g	Snout-Vent Length, cm	Head, cm	Heart Mass, g	Heart-to-Body Mass, g/g
Juveniles					
Incubated at 21% oxygen (*n* = 11)	1,399.64 ± 185.03	34.55 ± 1.36	8.74 ± 0.36	2.79 ± 0.28	0.0021 ± 0.0001
Incubated at 10% oxygen (*n* = 6)	741.92 ± 118.24*	29.04 ± 1.41*	7.62 ± 0.37	1.85 ± 0.29	0.0025 ± 0.0001*

Values are means ± SE. Morphometric measurements were taken in embryonic alligators at 90% development or 3-yr-old juveniles.

* Significant difference between oxygen exposure groups, one-way ANOVA, *P* ≤ 0.05.

#### Effects of ontogeny and developmental hypoxia on mitochondrial respiration.

OXPHOS was significantly greater in juvenile alligator cardiac mitochondria compared with their embryonic counterparts, regardless of which complexes were involved in electron transfer (Fig. 2). There were no significant effects of ontogeny on Leak respiration states (Fig. 3, *A–D**)*, but mitochondria from juveniles were more efficient than embryos, as attested by greater RCRs and P/O ratios with Complex I substrates (Fig. 3, *E–H*).

Developmental hypoxia had no effect on any respiratory parameter measured in embryonic alligator mitochondria (Figs. 2 and 3). Similarly, juvenile OXPHOS was similar between oxygen exposure groups (Fig. 2). However, Leak states (Leak_T_ and Leak_N_) with malate and pyruvate as substrates (electron transfer through Complexes I–IV) were significantly lower in juveniles previously exposed to developmental hypoxia, which led to a significantly greater RCR and a trend towards an increased P/O ratio (Fig. 3, left panels). There was also a tendency for this pattern to occur with succinate as a substrate (electron transfer through Complexes II–IV, Fig. 3, right panels), but this effect was not statistically resolvable. Leak respiration rate is predominantly controlled by proton leak and any contaminating ATPase activity (e.g., uncoupled ATPases). To remove the possibility of contamination, juvenile mitochondria from both exposure groups (*n* = 3 normoxia and 3 hypoxia) were treated with oligomycin, an inhibitor of the F_1_F_o_ ATPase, to induce Leak_Omy_. Oligomycin reduced Leak_T_ respiration rate in normoxic (by 77.2% ± 7.0) and hypoxic (by 83.7% ± 0.9) mitochondria. The difference in Leak respiration rates remained significant in the presence of oligomycin (normoxia 0.95 ± 0.19 and hypoxia 0.55 ± 0.03 nmol·min^−1^·mg^−1^; 1-way ANOVA, *P* = < 0.05), which indicates that the reduction in Leak_N_ and Leak_T_ respiration rates in juveniles previously exposed to developmental hypoxia is due to differences in intrinsic uncoupling processes.

#### Effects of ontogeny and developmental hypoxia on mitochondrial enzymatic activity.

**Fig. 4.**
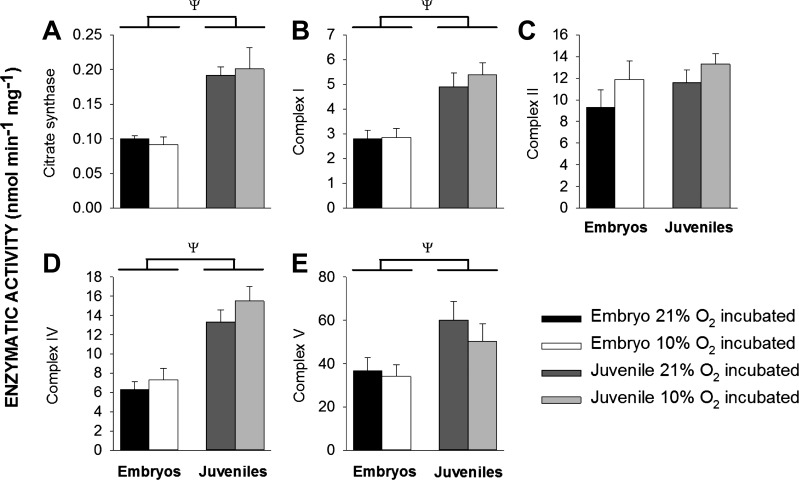
The effect of ontogeny and developmental hypoxia on mitochondrial enzymatic activity. Enzymatic activity was measured in homogenized cardiac tissue from alligator embryos exposed to 21% and 10% oxygen during development (black and white bars, respectively) and their juvenile counterparts (dark and light gray bars, respectively) raised in 21% oxygen. Citrate synthase (*A*), and Complex I, II, IV, and V of the electron transport chain (*B–E*) were assayed using spectrophotometry (details in methods). Data are means ± SE. ψSignificant difference between embryos and adults (2-way ANOVA). *P* < 0.05, *n* = 6 for all experimental groups.

The enhanced mitochondrial OXPHOS in juvenile vs. embryonic alligators was accompanied by enhanced enzymatic activities of Complexes I, IV, and V and a higher citrate synthase activity (Fig. 4). However, once the enzymatic activity of the complexes were normalized to citrate synthase activity there was no effect of ontogeny (data not shown). Developmental hypoxia had no effect on enzyme activities in either embryonic or juvenile American alligator mitochondria (Fig. 4).

## DISCUSSION

Hypoxia is a pervasive and powerful environmental stressor for many embryonic vertebrates, yet very little is known about the developmental plasticity of mitochondrial function in response to hypoxia. Developmental hypoxia induced growth restriction and increased relative heart mass in embryos, and this phenotype persisted into juvenile life. Contrary to our hypothesis, developmental hypoxia had no effect on mitochondrial function or enzymatic Complex activities in embryonic alligator hearts. However, juvenile alligators previously exposed to developmental hypoxia had more efficient mitochondria compared with their normoxic counterparts. Collectively, our results suggest developmental hypoxia can have life-long consequences for alligator morphology and metabolic function.

### 

#### Developmental hypoxia has lasting effects on alligator morphology.

Similar to previous studies, developmental hypoxia induced growth restriction and an increased heart-to-body weight ratio in alligator embryos (Table 1). Growth restriction and increased relative organ mass are common morphological signatures in a range of vertebrates subjected to developmental hypoxia [mammals (58, 60); birds (16, 27, 43, 50); reptiles (12, 18, 69)]. The relative increase in heart mass may be due to a suppression of somatic growth, a response of the cardiac tissue to hypoxia directly, or to a secondary response due to endocrine or hemodynamic changes in the embryonic system. Although our study cannot distinguish between these possibilities, recent work has identified discrete windows in alligator development where embryonic and cardiac growth are affected by hypoxia differentially (67). Cardiac enlargement results after hypoxic exposure between 20% and 40% of incubation, whereas embryonic growth is suppressed when hypoxia occurs from 70% to 90% of incubation (67). These results suggest developmental hypoxia increases relative cardiac mass in alligators due to cardiac enlargement and not suppression of somatic growth. The present study extends these findings further by demonstrating that the relative increase in cardiac mass and growth restriction associated with developmental hypoxia persists into juvenile life (Table 1). Given that cardiac mass is an index of stroke volume in ectotherms (35, 36); our results suggest developmental hypoxia may have lasting effects on cardiac performance in juvenile and adult alligators.

#### Alligator hearts undergo metabolic remodeling during development.

Mitochondria from alligators that developed in control conditions (i.e., not subjected to developmental hypoxia) were functionally different between embryos and juveniles. Compared with their embryonic counterparts, juvenile alligator mitochondria had higher rates of respiration and more efficient coupling between respiration and phosphorylation (Figs. 2 and 3). Furthermore, juvenile mitochondria had increased activities of citrate synthase (a proxy for mitochondrial density) and enzymatic activities of ETC Complexes (Fig. 3). However, once the enzymatic activities were normalized to citrate synthase activity (65), rather than total tissue protein content, the differences between embryos and juveniles disappeared. These results suggest ontogenic differences in oxidative capacity in alligators are mostly due to differences in mitochondrial density, rather than function. Nevertheless, we cannot rule out the possibility that the greater oxidative capacity reflects increased enzymatic activities within the mitochondria themselves. Future work could distinguish between these two possibilities by quantifying mitochondrial density with electron microscopy.

Structural and functional remodeling of mitochondria during development is a well-established phenomenon in mammals and is thought to occur in response to the growing energetic demands of development (54). Mammalian embryonic mitochondria undergo considerable structural and functional changes during midpregnancy coinciding with a switch from anaerobic glycolytic metabolism to oxidative phosphorylation (3). Nevertheless, the oxidative capacities of mammalian mitochondria from late-gestation fetuses are considerably lower than those from juvenile or adult mitochondria (51). After birth, the rise in ambient oxygen levels, metabolic rate, cardiac output, and energy demands requires an upregulation of mitochondrial biogenesis, a switch from glycolysis to β-oxidation of lipids (which generate larger amounts of ATP per unit of substrate), and activation of nuclear and mitochondrial gene expression (47, 51, 54). The mechanisms underlying postnatal metabolic remodeling in mammals are incompletely understood, but recent evidence suggests mitochondrial biogenesis is upregulated as a consequence of Pgc1α/β expression (46), and the switch in substrate utilization occurs via hypoxia-inducible factor (HIF) signaling (54). It remains to be investigated whether similar signaling mechanisms underlie the developmental metabolic switch in alligator hearts.

#### Developmental hypoxia had no effect on embryonic alligator mitochondrial function.

OXPHOS, Leak respiration, mitochondrial efficiency, and tissue enzymatic activities of Complexes I–IV were unaltered by developmental hypoxia in embryonic alligators (Figs. 2–4). This result suggests alligator mitochondria are relatively insensitive to large fluctuations in oxygen between 20 and 90% of embryonic development. To our knowledge, only three studies have investigated the effects of developmental hypoxia on vertebrate mitochondrial respiration. In mammalian models of prenatal hypoxia, fetuses from pregnant dams exposed to 10% oxygen have lower rates of respiration, mitochondrial efficiency, and enzymatic activities of ETC complexes, and these effects are interpreted as pathological (4, 11). In contrast, the efficiency of ATP production is increased in neuronal mitochondria from chick embryos exposed to 13% oxygen, and this effect is heightened in native highland species (6). This latter study suggests compensatory mechanisms exist in embryonic mitochondria from oviparous animals that allow them to adapt to hypoxic stress. Nevertheless, the present study was unable to detect any such differences in embryonic American alligators.

There are several possibilities for the lack of an embryonic mitochondrial response in our study. First, alligator embryos may compensate for hypoxia by enhancing oxygen extraction pathways to maintain mitochondrial oxygen tensions above critical levels. Such mechanisms may include a redistribution of blood flow to the heart, which is a common response to developmental hypoxia in mammals (27). Alternatively, exposing embryos to 10% ambient oxygen is not sufficient to inhibit OXPHOS or activate oxygen-sensitive metabolic pathways [e.g., HIF1α or AMPK (53, 61)]. In this regard, it is possible that mitochondrial oxygen dependence is lower in embryonic vs. adult mitochondria which would minimize cellular oxygen limitation and allow ATP production at low oxygen tensions. Nevertheless, the effects of ontogeny on mitochondrial P50s have not been measured in vertebrates. Last, anaerobic metabolism in hypoxic embryonic alligators may compensate for OXPHOS inhibition. The relative contribution of energy production by aerobic and anaerobic pathways is not known in embryonic alligators, but the well-recognized resistance of embryonic hearts to anoxia suggests that anaerobic pathways are robust at early life stages. Clearly, more research is necessary to understand the underlying mechanisms which permit the impressive hypoxia tolerance of alligator embryonic mitochondria.

#### Juvenile alligators previously exposed to developmental hypoxia have more efficient mitochondria.

In contrast to the embryos, we found significant differences between oxygen exposure groups in mitochondria from juvenile alligators. Leak respiration rates were lower and mitochondrial efficiency was higher in juvenile alligators from hypoxic incubations, compared with their normoxic counterparts. Furthermore, the lower Leak respiration rate is due to reduced proton leak, as the differences in juvenile leak state persisted in the presence of oligomycin. Proton leak within mitochondria is estimated to account for ~20% of whole animal oxygen consumption (14). Thus a significant proportion of the oxygen that an organism consumes does not result in ATP production. A reduction in Leak respiration allows greater mitochondrial coupling between oxidation and phosphorylation (exemplified by an increase in the RCR, Fig. 3*E*) resulting in a greater capacity for substrate oxidation and ATP turnover (10). The mechanism driving the reduced proton leak in juvenile alligators from hypoxic incubations was not determined in our study but may include decreased proton conductance of the inner mitochondrial membrane (possibly by changes in membrane composition or the expression of uncoupling proteins) or changes in mitochondrial membrane potential and proton motive force (14).

The concept that perturbations of the developmental environment can alter juvenile and adult phenotypes has now become known as “developmental programming” or the “Developmental Origins of Health and Disease, DOHAD” (28, 68). Some programmed changes in morphology and function are apparent in the prenatal period or immediately after birth, such as growth restriction and increased relative heart mass (Table 1). However, other programmed changes may only become evident later in life, often in association with aging or an additional environmental stress (22, 32, 68). The decrease in Leak respiration rate in juvenile, but not embryonic, alligators from hypoxic incubations may simply represent a latent effect of developmental hypoxia. Alternatively, increasing oxygen tension from 10% to 21% at 90% of development may represent a hyperoxic stress to the hypoxic embryos, thereby subjecting them to a secondary programming stimulus. Therefore, the decrease in Leak respiration may be a response to developmental hyperoxia, rather than hypoxia. To distinguish between these possibilities, it would be of interest to continue the hypoxic incubation beyond 90% development and raise the posthatch phenotypes to the juvenile stage under hypoxic conditions.

### Perspectives and Significance

The adaptive significance of the reduction in Leak respiration rate in juveniles from hypoxic incubations is an area for future research. One may expect strong evolutionary selection pressure exists to reduce mitochondrial proton leak and maximize mitochondrial efficiency. However, a trade-off exists between efficient ATP production and ROS generation (63). ROS production is an inevitable consequence of electron transport in the ETC, and it is an essential aspect of cell signaling. Nevertheless, excessive ROS generation can cause lipid peroxidation, DNA damage, apoptosis, and, consequently, whole organ and organismal failure (70). For this reason, the balance between ROS generation and ROS scavenging is a highly controlled process. ROS generation depends on mitochondrial membrane potential, so a decrease in Leak respiration could possibly increase ROS production (63). Therefore, alligators may incur significant energy savings from exposure to hypoxia during development, but basal ROS production could be elevated, which can have consequences for lifespan [“uncoupling to survive” hypothesis (8)]. Nevertheless, the mitochondrial membranes of ectothermic vertebrates are generally less leaky than mammals, and they possess robust antioxidant defenses (9). Perhaps the efficiency of ATP production is a priority for ectothermic vertebrates which regularly encounter hypoxic environments and are naturally protected from ROS. Clearly, more research is necessary to confirm whether exposure to developmental hypoxia confers a phenotypic or survival advantage for alligators. In this regard, it would be of interest to raise the posthatch phenotypes to the juvenile stage under conditions of intermittent hypoxia vs. normoxia and assess long-term growth rate and body size.

## GRANTS

This work was funded by a National Science Foundation (NSF) Career Award to D. A. Crossley II (IBN IOS-0845741), a NSF Award to E. M. Dzialowski. (IOS-1146758), and pilot funding from the Wellcome Trust to G. L. J. Galli and H. A. Shiels (Grant 105610/Z/14/Z).

## DISCLOSURES

No conflicts of interest, financial or otherwise, are declared by the author(s).

## AUTHOR CONTRIBUTIONS

G.L.G., R.M.E., H.A.S., and D.A.C. conception and design of research; G.L.G. and J.C. performed experiments; G.L.G. and J.C. analyzed data; G.L.G., J.C., E.M.D., and D.A.C. interpreted results of experiments; G.L.G. prepared figures; G.L.G. drafted manuscript; G.L.G., E.M.D., H.A.S., and D.A.C. edited and revised manuscript; G.L.G., J.C., R.M.E., E.M.D., H.A.S., and D.A.C. approved final version of manuscript.
